# Appointment in the Navy

**Published:** 1863-01

**Authors:** 


Appointment in the Navy.—Dr. J. F. Norton, of Fort Edward, N. Y., a frequent contributor to our pages, has received appointment as Surgeon United States Navy, and is to be detailed upon one of the new Iron-clad Steamers now nearly ready. We hope not to be deprived of his frequent contributions to our pages by this change in his field of observation.

